# Digital Eye Strain from Digital Device Usage Among University Students: Prevalence and Associated Factors

**DOI:** 10.3390/ijerph23050542

**Published:** 2026-04-22

**Authors:** Praphatson Sengsoon, Nattavipa Nuthong, Roongnapa Intaruk, Chalermsiri Theppitak, Orawan Yeampattanaporn, Netchanok Jianramas, Thanaporn Semphuet, Syarifah Fatima Yasmin

**Affiliations:** 1 Movement Sciences and Exercise Research Center, Walailak University (MoveSE-WU), Nakhon Si Thammarat 80160, Thailand; roongnapa.in@wu.ac.th; 2 Department of Physical Therapy, School of Allied Health Sciences, Walailak University, Nakhon Si Thammarat 80160, Thailand; nattavipa.nu@mail.wu.ac.th; 3 School of Occupational Health and Safety, Suranaree University of Technology, Nakhon Ratchasima 30000, Thailand; chalerm@sut.ac.th; 4 Department of Physical Therapy, Faculty of Physical Therapy, Srinakharinwirot University, Nakhonnayok 26120, Thailand; orawany@g.swu.ac.th; 5 Department of Physical Therapy, Faculty of Allied Health Sciences, Thaksin University, Phatthalung 93110, Thailand; netchanok.j@tsu.ac.th (N.J.); thanaporn.s@tsu.ac.th (T.S.); 6 Department of Physical Therapy, Faculty of Health and Sport Science, Universitas Negeri Makassar, Makassar 90222, Indonesia; syarifah.fatima@unm.ac.id

**Keywords:** digital eye strain, digital device usage, prevalence, associated factors, university students

## Abstract

**Highlights:**

**Public health relevance—How does this work relate to a public health issue?**

- Digital eye strain is highly prevalent among university students due to prolonged digital device use.
- This study highlights the impact of digital lifestyles on visual health in young adults.

**Public health significance—Why is this work of significance to public health?**

- Multiple modifiable factors, including behavioral, ergonomic, and environmental factors, are associated with digital eye strain.
- The findings provide important epidemiological evidence in a university population.

**Public health implications—What are the key implications or messages for practitioners, policy makers and/or researchers in public health?**

- Promoting healthy digital behaviors and improving environmental conditions may help reduce digital eye strain.
- The results support the development of preventive strategies and public health policies in the digital era.

**Abstract:**

**Objective:** To study the prevalence and associated factors of digital eye strain among university students. **Methodology:** A cross-sectional survey and analytical study was conducted on 387 university students, ranging from 1st to 4th year, aged 18–23 years. The participants were digital device users who had not been medically diagnosed with any eye diseases affecting their use of digital devices. Statistical analyses were performed using the Descriptive Statistics, Chi-square test, and Fisher’s exact test. **Results:** The prevalence of digital eye strain among university students was found to be 80.40%. The most common symptoms were headache (80.62%), burning sensation in the eyes (75.19%), and eye pain (71.06%). The study found that 30.49% were male and 69.51% were female, with an average age of 20.07 ± 0.07 years. It was found that gender (*p* < 0.05, Phi = 0.14), vision problems (*p* < 0.05, Phi = 0.20), wearing light-filtering glasses (*p* < 0.05, Phi = 0.12), average daily smartphone screen time (*p* < 0.05, Phi = 0.19), avoiding digital devices before sleep (*p* < 0.05, Phi = 0.22), glare (*p* < 0.05, Phi = 0.19), wind exposure to the eyes (*p* < 0.05, Phi = 0.20), and ambient air conditions (*p* < 0.05, Phi = 0.15) were significantly associated with digital eye strain (*p* < 0.05); however, the strength of these associations was small (Phi = 0.12–0.22), indicating limited practical impact. **Conclusions:** Digital eye strain is highly prevalent among university students. Although several factors were statistically associated with digital eye strain, the small effect sizes suggest that each factor contributes only modestly. These findings highlight the multifactorial nature of digital eye strain and the importance of considering combined behavioral, environmental, and ergonomic influences.

## 1. Introduction

Prolonged digital device use has been strongly associated with digital eye strain (DES), also known as Computer Vision Syndrome (CVS). The DES is defined as the development or exacerbation of recurrent ocular symptoms and/or signs specifically related to digital screen viewing. DES encompasses a broad range of visual and ocular symptoms, including dry eyes, blurred vision, frequent blinking, headaches, ocular discomfort, redness, itching, tearing, photophobia, difficulty focusing, eyelid heaviness, foreign body sensation, halos around lights, and diplopia [1,2,3]. High prevalence rates of DES have been reported among university students in several countries, including Egypt (87.9%) [4], Romania (86.1%) [5], India (77.5%) [6], Saudi Arabia (72.4%) [1], and China (63.5%) [7]. Overall, approximately 70–90% of university students worldwide experience symptoms related to prolonged digital device use [8].

Recent evidence from epidemiological studies and systematic reviews further highlights the global burden of digital eye strain (DES) among young adults. A systematic review and meta-analysis reported an overall prevalence of approximately 66–69%, with higher rates observed among university students, reaching up to 76.1% [9]. Individual studies conducted in different regions have also reported consistently high prevalence rates, such as 76.1% in Saudi Arabia [10], 68.5% in India [11], and over 70% in several university populations worldwide [12]. These findings are further supported by recent cross-sectional studies demonstrating high DES prevalence among university students in various settings, reinforcing the widespread nature of this condition [4,13,14].

In addition to its high prevalence, behavioral factors play a critical role in the development of digital eye strain. Prolonged screen exposure, particularly continuous use without adequate rest breaks, has been consistently identified as a major risk factor [15,16]. Excessive daily screen time, especially from smartphones, increases visual demand and reduces blink rate, leading to tear film instability and ocular surface discomfort [17]. Furthermore, screen use during nighttime, particularly before sleep, may exacerbate symptoms by disrupting circadian rhythms and impairing ocular recovery, partly due to blue light exposure affecting melatonin secretion [18].

Environmental and ergonomic conditions also contribute significantly to the occurrence and severity of digital eye strain. Factors such as screen glare, improper lighting, and low ambient humidity have been shown to increase visual discomfort and fatigue [17]. Direct airflow from fans or air conditioning may accelerate tear evaporation, further contributing to dry eye symptoms [19]. Moreover, suboptimal ergonomic conditions, including inappropriate viewing distance, screen positioning, and poor posture, may increase both visual strain and musculoskeletal load during prolonged digital device use [16].

Given the multifactorial nature of digital eye strain, several preventive strategies have been recommended. Behavioral modifications, such as limiting continuous screen time, increasing blink frequency, and adopting the 20-20-20 rule, are commonly suggested to reduce visual fatigue [13]. In addition, optimizing ergonomic conditions, including appropriate screen height, viewing distance, and lighting, may help minimize visual demand [17]. Environmental adjustments, such as reducing glare and maintaining adequate humidity, are also important for preserving ocular surface health [19]. However, despite these recommendations, adherence to preventive strategies remains inconsistent among university students.

Despite this growing body of evidence, the reported prevalence varies widely across studies, ranging from approximately 25% to over 90%, depending on differences in study populations, measurement tools, and patterns of digital device use [20]. These variations underscore the importance of context-specific investigations to better understand the determinants of DES in different populations.

The global evidence indicating a high prevalence of digital eye strain (DES), limited research has been conducted among university students in Thailand. Furthermore, while previous studies have largely focused on estimating prevalence, fewer investigations have systematically examined the behavioral, ergonomic, and environmental factors contributing to DES within this population. Given the high level of digital exposure among Thai young adults, identifying both the magnitude of DES and its associated risk factors is essential for developing targeted preventive and ergonomic intervention strategies.

Although several validated instruments for assessing DES, such as the Computer Vision Syndrome Questionnaire (CVS-Q), have been widely used in previous studies, important gaps remain in the literature. In particular, most studies have primarily focused on prevalence or isolated risk factors, with limited integration of multiple domains influencing DES. Moreover, there is a scarcity of studies that comprehensively evaluate these multidimensional factors among university students in Thailand.

Therefore, this study aimed to determine the prevalence of digital eye strain and to identify factors associated with DES among university students. The findings of this study are expected to provide foundational epidemiological data and inform preventive measures to reduce digital device–related ocular strain in higher education settings.

## 2. Materials and Methods

### 2.1. Study Population

This study employed a survey-based design with a cross-sectional analytical approach to investigate the prevalence of digital eye strain (DES) and its associated factors among university students. The study was conducted in accordance with the Declaration of Helsinki and approved by the Institutional Review Board (Ethics Committee) of Walailak University (WUEC-25-113-01, April 2025). All participants provided written informed consent prior to participation. The study population consisted of undergraduate students enrolled at Walailak University. Inclusion criteria were: (1) students in years 1–4 of undergraduate study, aged between 18 and 23 years; (2) regular use of digital devices (e.g., smartphones, computers, or tablets); and (3) ability to read and comprehend Thai. Exclusion criteria included a prior medical diagnosis of ocular or neurological conditions that could independently affect visual function or digital device use, such as diabetic retinopathy, glaucoma, cataract, increased intracranial pressure, macular degeneration, or optic neuritis [21].

The required sample size was calculated using Taro Yamane’s formula for finite populations: n = N/(1 + Ne^2^), where n is the required sample size, N is the total population size, and e is the margin of error. Based on the total number of undergraduate students meeting the study criteria at Walailak University and a margin of error of 5%, the minimum required sample size was estimated to be 387 participants. To enhance representativeness, the final sample was proportionally allocated according to academic year as follows: 109 first-year students (28.23%), 96 second-year students (24.94%), 100 third-year students (25.72%), and 82 fourth-year students (21.10%).

### 2.2. Procedure

Data were collected using a structured self-administered questionnaire, which was distributed online through Google Forms. The questionnaire consisted of seven dimensions: (1) participant screening information; (2) personal factors (e.g., sex, age, visual problems, and use of corrective lenses); (3) digital device characteristics (e.g., type of device, viewing distance, screen position, and screen settings); (4) behavioral factors (e.g., duration of screen use, rest breaks, and screen time patterns); (5) environmental factors (e.g., lighting conditions, glare, airflow, and ambient air conditions); (6) knowledge related to the prevention of digital eye strain (DES); and (7) frequency and severity of DES symptoms. This multidimensional structure allowed for a comprehensive assessment of factors associated with digital eye strain. The Thai version of the questionnaire, including detailed items and scoring procedures, is provided in the Supplementary Materials (Supplementary File S1).

Digital eye strain (DES) symptoms were assessed using the Computer Vision Syndrome Questionnaire (CVS-Q), a validated instrument that evaluates both the frequency and intensity of ocular symptoms associated with digital device use. Each symptom score was calculated as the product of frequency and intensity and subsequently recoded according to the standard scoring system.

For use in this study, the questionnaire was translated into Thai and evaluated for content validity by three experts, including two specialists in ergonomics and one specialist in occupational health and safety. The Index of Item–Objective Congruence (IOC) was applied, with an acceptable threshold set at ≥0.50, and the questionnaire was revised according to expert recommendations.

Prior to the main data collection, a pilot test was conducted with 10 university students who regularly used digital devices but were not included in the study sample to assess clarity, comprehensibility, and appropriateness. A second pilot test was then conducted with 30 university students to further evaluate clarity, appropriateness, and inter-rater agreement. Following these refinements, the finalized questionnaire was distributed online for data collection, and the collected data were subsequently subjected to statistical analysis [22].

Knowledge on the prevention of digital eye strain was assessed using five items covering key preventive practices, including appropriate screen use, regular rest breaks, environmental adjustments, and visual health behaviors. Each correct response was scored as 1 point, resulting in a total score ranging from 0 to 5, with higher scores indicating greater knowledge. The items were developed based on established recommendations for reducing digital eye strain.

Digital eye strain (DES) was assessed using the Computer Vision Syndrome Questionnaire (CVS-Q). Symptom scores were calculated as the product of frequency and intensity and subsequently recoded according to the standard scoring system. A total score of ≥6 was considered indicative of DES, with severity classified as mild (6–12), moderate (13–18), and severe (19–32), based on the original validation study [2].

### 2.3. Statistical Analysis

All statistical analyses were performed using IBM SPSS Statistics for Windows, Version 26.0 (IBM Corp., Armonk, NY, USA). Statistical significance was set at *p* < 0.05. Descriptive statistics were used to summarize participant characteristics and study variables. Frequencies and percentages were calculated for categorical variables, while means and standard deviations were reported for continuous variables. The prevalence of digital eye strain (DES) was determined and expressed as a percentage.

Associations between DES and potential risk factors—including personal factors, digital device characteristics, behavioral factors, and environmental factors—were examined using the Chi-square test. Fisher’s exact test was applied when expected cell counts were less than five.

## 3. Result

A total of 387 university students participated in this study. Data were collected via an online questionnaire between 7 August and 11 August 2025. The response rate was 100%.

### 3.1. Personal Factors

The personal characteristics of the participants are presented in Table 1.

### 3.2. Digital Device Characteristics

From the survey on digital device characteristics among university students, the devices included desktop computers, laptops, tablets/iPads, smartphones, and others such as Apple Watches, smartwatches, and pen displays. The findings revealed that the most commonly used digital device was the smartphone (n = 382, 98.70%), followed by tablets/iPads (n = 356, 91.99%) and laptops (n = 218, 56.33%), respectively.

Most participants had a viewing distance between the desktop computer (55.20%) or laptop (58.26%) screens and their eyes of approximately one arm’s length, with the screen positioned at eye level. In contrast, the viewing distance for tablet/iPad (55.90%) and smartphone (53.14%) use was approximately 30 cm, with the screens positioned below eye level.

In addition, all devices were used with eye protection mode (dark mode) and were covered with either clear film or tempered glass. Most participants also reported adjusting screen brightness and font size appropriately.

### 3.3. Behavioral Factors

The behavioral factors related to digital device usage among university students are presented in Table 2. In addition, the eye rest behaviors of university students, including rest frequency and duration, are summarized in Table 3.

### 3.4. Environmental Factors

The environmental factors related to digital device use among university students are presented in Table 4.

### 3.5. Prevalence of Digital Eye Strain

**Figure 1:**
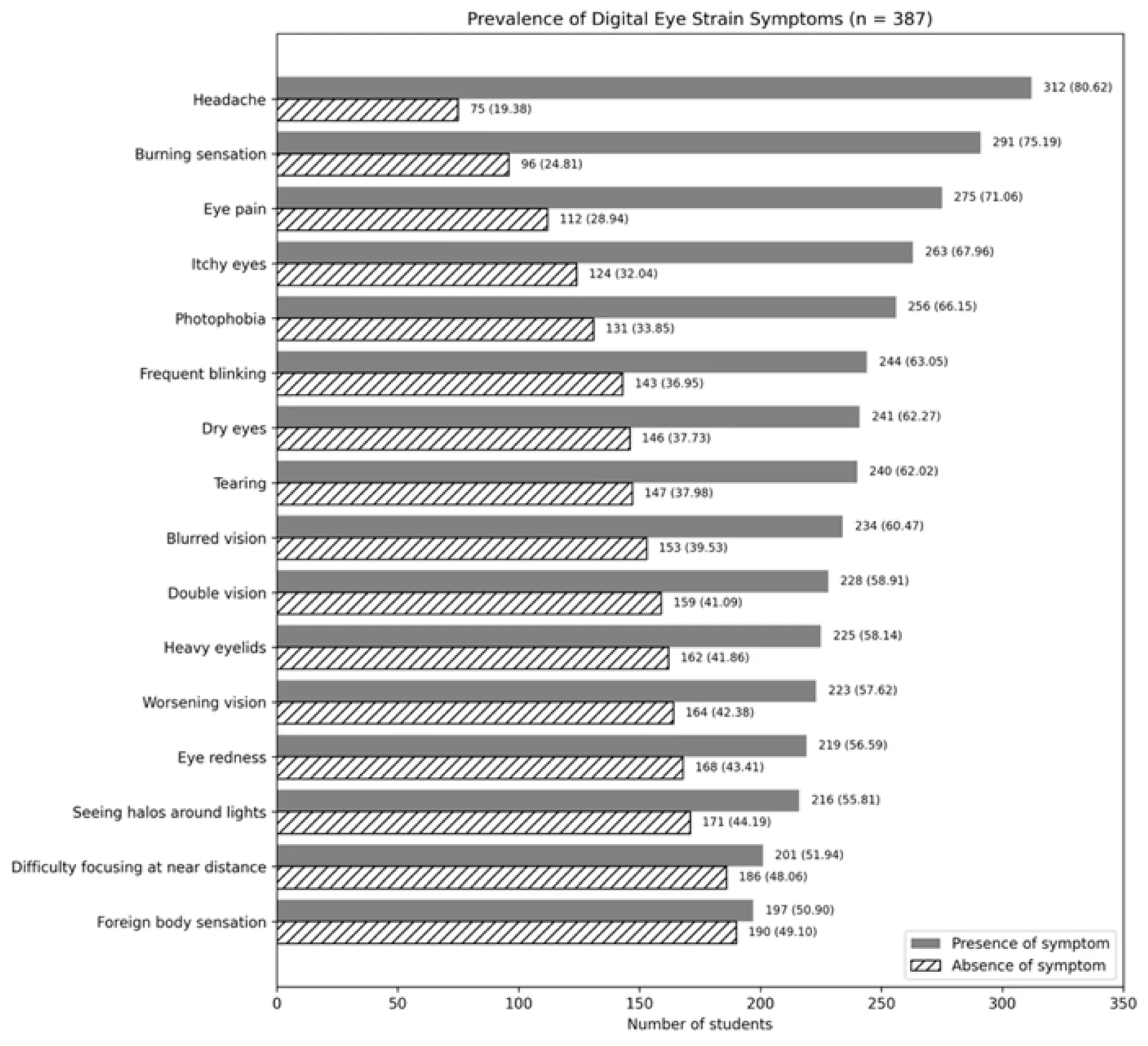
Prevalence of individual digital eye strain symptoms among university students (n = 387).

Based on the defined criteria, 311 students (80.40%) were classified as having digital eye strain, while 76 students (19.60%) were classified as not having digital eye strain. The most frequently reported symptoms were headache (312 students, 80.62%), burning sensation (291 students, 75.19%), and eye pain (275 students, 71.06%), as shown in Figure 1.

### 3.6. Association Between Factors and Digital Eye Strain

The strength of associations was interpreted using Phi coefficients, where values of approximately 0.10, 0.30, and 0.50 represent small, medium, and large effect sizes, respectively. In this study, the significant associations observed (Phi = 0.12–0.22) indicated small to modest effect sizes. The factors associated with digital eye strain are presented in Table 5.

## 4. Discussion

### 4.1. Prevalence of Digital Eye Strain Among University Students

From the survey of 387 university students, 311 students (80.40%) were classified as having digital eye strain (DES), while 76 students (19.60%) did not have DES. The most commonly reported symptoms were headache (312 students, 80.62%), burning sensation (291 students, 75.19%), and eye pain (275 students, 71.06%).

The prevalence observed in this study is relatively high compared with findings reported in other university populations. Previous studies have reported prevalence rates ranging from approximately 50% to 90% among university students and young adults. For example, a study conducted among university students in Riyadh, Saudi Arabia reported a prevalence of 76.1%, with headache being the most commonly reported symptom.

The higher prevalence observed in the present study may be attributed to several factors. Increased screen exposure, particularly prolonged smartphone use, may contribute to a greater risk of ocular symptoms. In addition, academic workload and online learning environments may lead to extended periods of near work without adequate rest breaks. Environmental and ergonomic factors, such as suboptimal lighting conditions, inappropriate viewing distance, and screen positioning, may further exacerbate digital eye strain.

These findings are consistent with previous studies indicating that prolonged visual engagement with digital devices, particularly at near distances, increases accommodative demand and continuous binocular coordination, leading to eye muscle fatigue and associated symptoms such as headache [10].

### 4.2. Association Between Personal Factors and Digital Eye Strain

The findings of this study showed that sex was significantly associated with digital eye strain (*p* < 0.05). Although several factors were significantly associated with digital eye strain, the magnitude of these associations was generally small to moderate, the small effect sizes observed in this study suggest limited clinical impact when factors are considered individually, reinforcing the importance of multifactorial assessment. This suggests that digital eye strain is influenced by multiple interacting behavioral, ergonomic, and environmental factors rather than a single dominant determinant, highlighting the need for a comprehensive approach to prevention.

The previous study reported that females tend to use mobile phones more frequently and spend longer periods looking at screens for entertainment and communication activities [23]. The visual problems were significantly associated with digital eye strain (*p* < 0.05). Visual problems are an important factor related to eye strain, particularly from continuous digital device use. Sheppard et al. (2018) reported that digital eye strain results from prolonged near focusing combined with abnormalities in accommodation and convergence, leading to symptoms such as eye pain, blurred vision, and burning sensation [24].

Furthermore, this study found that wearing glasses with blue light filtering properties was significantly associated with digital eye strain (*p* < 0.05). However, this finding should be interpreted with caution, as it does not indicate a causal relationship. It is possible that individuals experiencing digital eye strain are more likely to use blue light filtering glasses in an attempt to reduce their symptoms, suggesting potential reverse causation rather than a direct effect of blue light exposure.

The role of blue light filtering in reducing digital eye strain remains controversial. Although blue light emitted from digital screens has been suggested to contribute to ocular discomfort and potential retinal stress [25,26], evidence regarding its effectiveness in alleviating digital eye strain symptoms remains inconsistent. Instead, digital eye strain is more strongly associated with factors such as reduced blink rate, tear film instability, and prolonged near work.

### 4.3. Association Between Digital Device Characteristics and Digital Eye Strain

This study found that digital device characteristics were not significantly associated with digital eye strain (*p* > 0.05). The few studies supported this concept, indicating that eye strain does not result from a single factor but rather from multiple complex factors. These include usage behaviors, such as prolonged digital device use without taking breaks and reduced blink rate. Such behaviors cause the eye muscles to work continuously for near focusing and also accelerate ocular surface dryness, which are the primary causes of digital eye strain [3,16].

### 4.4. Association Between Behavioral Factors of Digital Device Use and Digital Eye Strain

The duration of smartphone use and average daily screen time were significantly associated with digital eye strain (*p* < 0.05). In addition, avoiding digital device use within 1–2 h before bedtime was also significantly associated with digital eye strain (*p* < 0.05). These findings are consistent with previous evidence indicating that prolonged screen exposure and excessive smartphone use are important behavioral risk factors for digital eye strain. Continuous use of digital devices increases visual demand and is associated with a reduced blink rate, leading to tear film instability and ocular surface dryness. Furthermore, screen exposure before bedtime may negatively affect sleep quality and circadian rhythm. Blue light emitted from digital screens has been shown to suppress melatonin secretion, a hormone that regulates sleep–wake cycles, thereby disrupting normal sleep patterns. Insufficient sleep and inadequate ocular recovery may increase susceptibility to digital eye strain and exacerbate symptom severity on the following day [27].

These findings highlight the importance of modifying behavioral factors, including limiting prolonged screen time and reducing digital device use before bedtime, as key strategies for preventing digital eye strain.

### 4.5. Association Between Environmental Factors and Digital Eye Strain

Environmental and ergonomic factors also play an important role in the development of digital eye strain. In this study, screen glare was significantly associated with digital eye strain (*p* < 0.05), consistent with previous findings indicating that glare reduces visual contrast and increases visual demand, leading to ocular discomfort and fatigue [24].

In addition, direct airflow from a fan or air conditioner blowing toward the eyes was significantly associated with digital eye strain (*p* < 0.05). This may be explained by increased tear evaporation and reduced tear film stability, resulting in symptoms such as dry eyes, burning sensation, and irritation during prolonged digital device use [28].

Furthermore, ambient environmental conditions, including air quality and humidity, were also significantly associated with ocular health (*p* < 0.05). Previous studies have suggested that maintaining appropriate environmental conditions, particularly adequate humidity, can help preserve tear film integrity and reduce the risk of ocular surface damage associated with prolonged screen exposure [16]. In addition, inappropriate lighting conditions and suboptimal workstation ergonomics, such as improper screen position and viewing angle, may further contribute to visual strain by increasing visual effort and postural discomfort during prolonged digital device use.

These findings highlight the importance of optimizing environmental and ergonomic conditions, including reducing glare, controlling airflow direction, and maintaining appropriate humidity, to minimize the risk of digital eye strain, particularly in prolonged digital work environments.

From a practical perspective, the findings of this study highlight several strategies that may help reduce the risk of digital eye strain. These include adopting the 20-20-20 rule (taking a 20 s break every 20 min to look at an object 20 feet away), optimizing ergonomic screen positioning (e.g., maintaining appropriate viewing distance and screen height), and performing regular blinking exercises to maintain tear film stability. In addition, limiting continuous screen exposure and reducing digital device use before bedtime may help prevent visual fatigue and improve ocular comfort. These strategies may be particularly beneficial for university students engaged in prolonged digital device use.

## 5. Conclusions

### 5.1. Summary of Findings

This study investigated the prevalence and associated factors of digital eye strain among 387 university students. The findings revealed that 311 students (80.40%) experienced digital eye strain, while 76 students (19.60%) did not. The most frequently reported symptoms were headache in 312 students (80.62%), burning sensation in 291 students (75.19%), and eye pain in 275 students (71.06%).

The results of this study provide important evidence regarding the prevalence and factors associated with digital eye strain among university students. Although several factors were statistically associated with digital eye strain, the magnitude of these associations was small, suggesting that each factor individually contributes only modestly to the overall risk. This supports the concept that digital eye strain is a multifactorial condition influenced by the combined effects of behavioral, environmental, and individual factors.

These findings may increase students’ awareness of digital eye strain and its potential impact. The data obtained can be utilized to develop preventive strategies, guide appropriate management approaches, and promote ocular health among students experiencing these symptoms. Furthermore, the baseline information generated from this study may serve as a foundation for future in-depth research.

### 5.2. Limitations and Recommendations for Future Research

#### 5.2.1. Limitations

This study has several limitations. First, the use of self-reported questionnaires to assess digital device use and digital eye strain (DES) may introduce measurement bias and response inaccuracies. In particular, the reliance on self-reported data without objective clinical assessment may lead to misclassification of the presence and severity of ocular symptoms.

In addition, the use of an online questionnaire (Google Forms) limited control over the conditions under which participants completed the survey, including the timing and environment of responses, which may have affected data reliability.

Another limitation of this study is the use of bivariate analysis without multivariate adjustment. As a result, potential confounding factors were not controlled, and the observed associations may be influenced by underlying variables not accounted for in the analysis. Therefore, the findings should be interpreted with caution, particularly regarding their generalizability and the ability to infer independent effects of each factor.

Despite these limitations, this study provides important baseline evidence on the prevalence and associated factors of digital eye strain among university students.

#### 5.2.2. Recommendations for Future Research

Future research should incorporate objective clinical assessments (e.g., ophthalmic examinations, tear film stability, blink rate, or accommodative function) to validate self-reported symptoms and improve diagnostic accuracy. In addition, future studies should employ multivariate analytical approaches, such as multiple logistic regression, to better account for confounding variables and strengthen the validity of the findings. Furthermore, longitudinal or experimental study designs are recommended to establish causal relationships and evaluate the effectiveness of targeted interventions, such as screen time regulation, ergonomic adjustments, and environmental modifications.

Moreover, mixed-method approaches, including qualitative interviews, may provide deeper insights into behavioral patterns and contextual factors influencing digital eye strain.

## Figures and Tables

**Table 1:** Personal Factors (n = 387).

Personal Factors	Number (Percentage)	Mean (SD)	Median (Min., Max.)
Sex (persons)			
Male	118 (30.49)		
Female	269 (69.51)		
Age (years)		20.07 (1.38)	20.00 (18.00, 23.00)
18	63 (16.30)		
19	86 (22.20)		
20	68 (17.60)		
21	108 (27.90)		
22	54 (14.00)		
23	8 (2.10)		
Year of study (academic year)			
Year 1	109 (28.23)		
Year 2	96 (24.94)		
Year 3	100 (25.72)		
Year 4	82 (21.10)		
School/Faculty			
Allied Health Sciences	90 (23.30)		
Public Health	21 (5.40)		
Informatics	43 (11.10)		
Pharmacy	18 (4.70)		
Medicine	28 (7.20)		
Management	16 (4.10)		
Accounting and Finance	35 (9.00)		
Law	9 (2.30)		
Nursing	5 (1.30)		
Political Science and Public Administration	10 (2.60)		
Science	22 (5.70)		
Engineering and Technology	25 (6.50)		
Liberal Arts	39 (10.10)		
Education	19 (4.90)		
Architecture and Design	4 (1.00)		
Veterinary Medicine	3 (0.80)		
Visual problems			
No visual problems	206 (53.20)		
Have visual problems	181 (46.80)		
- Myopia	96 (24.80)		
- Hyperopia	1 (0.30)		
- Astigmatism	14 (3.60)		
- Myopic astigmatism	69 (17.80)		
- Hyperopic astigmatism	1 (0.30)		
Wearing glasses			
Do not wear	279 (72.10)		
Wear	108 (27.90)		
Glasses with blue light filtering property			
No	193 (49.90)		
Yes	194 (50.10)		
Contact lens use			
Do not use	338 (87.30)		
Use	49 (12.70)		
Frequency of contact lens use			
- Less than 3 days per week	26 (6.70)		
- 3–5 days per week	13 (3.40)		
- 6–7 days per week	10 (2.60)		
Number of hours per day of contact lens use			
- Less than 6 h	14 (3.60)		
- 6–8 h	19 (4.90)		
- More than 8 h	16 (4.10)		
Artificial tear use			
Do not use	348 (89.90)		
Use	39 (10.10)		
Frequency of artificial tear use			
- 3–5 days per week	33 (8.50)		
- 6–7 days per week	6 (1.60)		
Knowledge on prevention of eye strain		4.24 (0.94)	4.00 (0.00, 5.00)
0 points	1 (0.30)		
1 point	5 (1.30)		
2 points	18 (4.70)		
3 points	40 (10.30)		
4 points	134 (34.60)		
5 points	189 (48.80)		

**Table 2:** Behavioral factors of digital device usage.

**Behavioral Factors for Desktop Computer Use (n = 105)**	**Number (Percentage)**
Duration of continuous desktop screen viewing per session	
Less than 2 h/session	34 (32.38)
2–4 h/session	42 (40.00)
4–6 h/session	19 (18.10)
More than 6 h/session	10 (9.52)
Average daily desktop screen viewing time	
Less than 2 h/day	18 (17.14)
2–4 h/day	39 (37.14)
4–6 h/day	23 (21.91)
6–8 h/day	14 (13.33)
More than 8 h/day	11 (10.48)
**Behavioral factors for laptop use (n = 218)**	**Number (Percentage)**
Duration of continuous laptop screen viewing per session	
Less than 2 h/session	100 (45.87)
2–4 h/session	78 (35.78)
4–6 h/session	34 (15.60)
More than 6 h/session	6 (2.75)
Average daily laptop screen viewing time	
Less than 2 h/day	50 (22.94)
2–4 h/day	74 (33.94)
4–6 h/day	55 (25.23)
6–8 h/day	20 (9.17)
More than 8 h/day	19 (8.72)
**Behavioral factors for tablet/iPad use (n = 356)**	**Number (Percentage)**
Duration of continuous tablet/iPad screen viewing per session	
Less than 2 h/session	86 (24.16)
2–4 h/session	181 (50.84)
4–6 h/session	56 (15.73)
More than 6 h/session	33 (9.27)
Average daily tablet/iPad screen viewing time	
Less than 2 h/day	13 (3.65)
2–4 h/day	46 (12.92)
4–6 h/day	71 (19.94)
6–8 h/day	98 (27.53)
More than 8 h/day	128 (35.96)
**Behavioral factors for smartphone use (n = 382)**	**Number (Percentage)**
Duration of continuous smartphone screen viewing per session	
Less than 2 h/session	152 (39.79)
2–4 h/session	147 (38.48)
4–6 h/session	47 (12.30)
More than 6 h/session	36 (9.43)
Average daily smartphone screen viewing time	
Less than 2 hours/day	20 (5.24)
2–4 h/day	62 (16.23)
4–6 h/day	84 (21.99)
6–8 h/day	80 (20.94)
More than 8 h/day	136 (35.60)
**Behavioral factors for other digital devices (n = 3)**	**Number (Percentage)**
Duration of continuous screen viewing per session	
Less than 2 h/session	3 (100)
2–4 h/session	0 (0)
4–6 h/session	0 (0)
More than 6 h/session	0 (0)
Duration of screen viewing within 1 day	
Less than 2 h/day	1 (33.33)
2–4 h/day	1 (33.33)
4–6 h/day	1 (33.33)
6–8 h/day	0 (0)
More than 8 hours/day	0 (0)

**Table 3:** Behavioral factors for eye rest.

**Resting Time (n = 387)**	**Number (Percentage)**
Do not rest	70 (18.10)
Rest	317 (81.90)
Duration of rest before returning to screen again	
Less than 30 min	194 (50.12)
30–60 min	79 (20.41)
60–90 min	14 (3.62)
More than 90 min	30 (7.75)
**Avoiding digital device use before bedtime (n = 387)**	**Number (Percentage)**
Do not avoid	305 (78.80)
Avoid	82 (21.20)
**Sleep duration (n = 387)**	**Number (Percentage)**
Less than 4 h	9 (2.30)
Approximately 4–5 h	120 (31.00)
Approximately 6–7 h	233 (60.20)
More than 8 h	25 (6.50)

**Table 4:** Environmental factors (n = 387).

Environmental Factors	Number (Percentage)
Adjustment of the environment while using digital devices	
Do not adjust the environment appropriately	53 (13.70)
Adjust the environment appropriately	334 (86.30)
Presence of screen glare	
None	51 (13.20)
Sometimes	310 (80.10)
Regularly	26 (6.70)
Direct airflow from a fan or air conditioner to the eyes while using digital devices	
None	95 (24.50)
Sometimes	233 (60.20)
Regularly	59 (15.20)
Air condition in the area where digital devices are used	
Very dry (dry enough to feel burning sensation in the nose/throat)	9 (2.30)
Slightly dry	63 (16.30)
Normal	296 (76.50)
Slightly humid	18 (4.70)
Very humid	1 (0.30)

**Table 5:** Factors associated with digital eye strain.

**Personal Factors**	***p*** **-Value**
Sex	0.02 * (Phi = 0.14)
Age	0.20
Year of study	0.70
School/Faculty	0.46
Visual problems	*p* < 0.01 ** (Phi = 0.20)
Type of refractive error	0.44 ^a^
Wearing glasses	0.23
Glasses with blue light filtering property	0.02 * (Phi = 0.12)
Contact lens use	0.53
Frequency of contact lens use	0.88 ^a^
Number of hours per day of contact lens use	0.26 ^a^
Artificial tear use	0.26
Frequency of artificial tear use	0.57 ^a^
Knowledge on prevention of eye strain	0.20 ^a^
**Digital Device Characteristics**	***p*** **-value**
Desktop computer	0.12
- Viewing distance between screen and eyes	0.33
- Eye level	0.56 ^a^
- Use of eye protection mode	0.16
- Use of Dark mode	0.87
- Screen protector installation	0.85 ^a^
Laptop	0.41
- Viewing distance between screen and eyes	0.42
- Eye level	0.20 ^a^
- Use of eye protection mode	0.52
- Use of Dark mode	0.77
- Screen protector installation	0.38
Tablet/iPad	0.97
- Viewing distance between screen and eyes	0.87
- Eye level	0.24 ^a^
- Use of eye protection mode	0.15
- Use of Dark mode	0.96
- Screen protector installation	0.69
Smartphone	0.05 ^a^
- Viewing distance between screen and eyes	0.13
- Eye level	0.07 ^a^
- Use of eye protection mode	0.16
- Use of Dark mode	0.66
- Screen protector installation	0.83 ^a^
Other digital devices	1.00 ^a^
- Viewing distance between screen and eyes	1.00 ^a^
- Eye level	1.00 ^a^
- Use of eye protection mode	1.00 ^a^
- Use of Dark mode	1.00 ^a^
- Screen protector installation	1.00 ^a^
Adjustment	
- Screen brightness	0.32 ^a^
- Font size	0.69
**Behavioral Factors**	***p*** **-value**
Duration of continuous desktop screen viewing per session	0.88 ^a^
Average daily desktop screen viewing time	0.88 ^a^
Duration of continuous laptop screen viewing per session	0.47 ^a^
Average daily laptop screen viewing time	0.22
Duration of continuous tablet/iPad screen viewing per session	0.66
Average daily tablet/iPad screen viewing time	0.11
Duration of continuous smartphone screen viewing per session	0.58
Average daily smartphone screen viewing time	0.01 ** (Phi = 0.19)
Duration of continuous other digital device screen viewing per session	***
Average daily other digital device screen viewing time	***
Eye rest	0.28
Duration of rest before returning to screen again	0.51
Non-digital activities	0.18
Avoiding digital device use before bedtime	*p* < 0.01 ** (Phi = 0.22)
Sleep duration	0.21
**Environmental Factors**	***p*** **-value**
Adjustment of environment while using digital devices	0.37
Presence of screen glare	*p* < 0.01 ** (Phi = 0.19)
Direct airflow from fan or air conditioner to the eyes	*p* < 0.01 ** (Phi = 0.20)
Air condition in the area where digital devices are used	0.04 ^a^* (Phi = 0.15)

Notes: * *p* < 0.05, ** *p* < 0.01, *** Statistical analysis could not be performed due to data being in a single category only, ^a^ Fisher’s Exact Test due to violation of Chi-square assumptions (E_ij_ < 5 in more than 20%).

## Data Availability

The data presented in this study are available on request from the corresponding author.

## Supplementary Materials

The following supporting information can be downloaded at: https://www.mdpi.com/article/10.3390/ijerph23050542/s1, Supplementary File S1: Questionnaire used in this study.
