# Supplementary material for: Digital Eye Strain from Digital Device Usage Among University Students: Prevalence and Associated Factors
Source: Int J Environ Res Public Health. 2026 Apr 22;23(5):542. doi: 10.3390/ijerph23050542 (PMC13205559; doi:10.3390/ijerph23050542)
Supplement: Supplementary file 1 [file ijerph-23-00542-s001.zip › ijerph-4200945-supplementary.pdf]

Supplementary File S1.

Questionnaire (English Version)

Title

Digital Eye Strain from Digital Device Usage among University Students: Prevalence and Associated Factors

Instructions

This questionnaire aims to investigate the prevalence and associated factors of digital eye strain among university students. The questions include factors related to digital device usage and the frequency and severity of eye strain symptoms.

All information provided will be kept confidential and used solely for research purposes. No personal identifiable information will be disclosed.

Please select the most appropriate answer or fill in the blank based on your actual behavior over the past 1 month.

Screening Questions

1. Are you a student at Walailak University?
   ☐ Yes ☐ No (Terminate)
2. Are you aged between 18–23 years?
   ☐ Yes ☐ No (Terminate)
3. Do you use digital devices (e.g., smartphone, laptop, tablet)?
   ☐ Yes ☐ No (Terminate)
4. Have you ever been diagnosed with any eye disease affecting digital device use (e.g., diabetic retinopathy, glaucoma, cataract, retinal degeneration, optic neuritis)?
   ☐ No ☐ Yes (Terminate)

Section 1: Personal Factors

1.1 Sex
☐ Male ☐ Female ☐ Other

1.2 Age
☐ 18 ☐ 19 ☐ 20 ☐ 21 ☐ 22 ☐ 23

1.3 Year of Study
☐ Year 1 ☐ Year 2 ☐ Year 3 ☐ Year 4

1.4 School/Faculty
(Select one)
☐ Allied Health Sciences
☐ Public Health
☐ Informatics
☐ Agricultural Technology and Food Industry
☐ Pharmacy
☐ Medicine
☐ Management
☐ Accounting and Finance
☐ Law
☐ Nursing
☐ Liberal Arts
☐ Political Science
☐ Science
☐ Engineering and Technology
☐ Education
☐ Architecture and Design

1.5 Refractive Errors (Multiple answers allowed)
☐ None
☐ Myopia
☐ Hyperopia
☐ Astigmatism
☐ Unsure

1.6 Use of Corrective Lenses
☐ No (no vision problem)
☐ No (despite having vision problem)
☐ Yes (inappropriate correction)
☐ Yes (appropriate correction)

1.7 Blue-Light Filtering Glasses
☐ No
☐ Yes (without filtering function)
☐ Yes (with filtering function)

1.8 Contact Lens Use
☐ No (Skip to 1.9)
☐ Yes

- Days per week: ______
- Hours per day: ______

1.9 Artificial Tears Use
☐ No (Skip to 1.10)
☐ Yes

- Days per week: ______
- Times per day: ______

1.10 Sleep Duration per Day
☐ <4 hours ☐ 4–5 hours ☐ 6–7 hours ☐ >8 hours

Section 2: Digital Device Usage and Behavioral Factors

2.1 Devices Used (Multiple answers allowed)
☐ Desktop (screen size: ___)
☐ Laptop (screen size: ___)
☐ Tablet/iPad (screen size: ___)
☐ Smartphone (screen size: ___)
☐ Others: ______

2.2 Use of Eye Comfort Mode (per device)
☐ Yes ☐ No

2.3 Use of Dark Mode (per device)
☐ Yes ☐ No

2.4 Screen Protector Type
☐ None ☐ Clear ☐ Matte

2.5 Continuous Screen Use per Session (per device)

2.6 Total Daily Screen Time (per device)

2.7 Viewing Distance (Computer/Laptop)
☐ <50 cm
☐ 51–70 cm
☐ >70 cm

2.8 Viewing Distance (Smartphone/Tablet)
☐ <30 cm
☐ 31–40 cm
☐ >41 cm

2.9 Screen Position (Computer/Laptop)
☐ Below eye level
☐ At eye level
☐ Above eye level

2.10 Screen Position (Smartphone/Tablet)
☐ Below eye level
☐ At eye level
☐ Above eye level

2.11 Eye Breaks During Use
☐ No
☐ Yes → Duration: ______

2.12 Non-digital Activities (e.g., outdoor activity)
☐ No ☐ Yes

2.13 Avoid Device Use Before Bedtime (1–2 hours)
☐ No ☐ Yes

2.14 Adjust Screen Brightness to Environment
☐ No ☐ Yes

2.15 Adjust Font Size for Comfortable Reading
☐ No
☐ Always
☐ Sometimes

2.16 Adjust Environment (e.g., reduce glare)
☐ No ☐ Yes

Section 3: Environmental Factors

3.1 Screen Glare from Lighting
☐ No ☐ Yes

3.2 Airflow Toward Eyes (fan/air-conditioner)
☐ None ☐ Sometimes ☐ Always

3.3 Ambient Air Condition
☐ Very dry ☐ Slightly dry ☐ Normal ☐ Slightly humid ☐ Very humid

---

Section 4: Knowledge on Digital Eye Strain Prevention

4.1 What is the 20-20-20 rule?
☐ Look 20 feet away for 20 seconds every 20 minutes
☐ Blink 20 times every 20 minutes
☐ Drink water every 20 minutes
☐ Rest 20 minutes after working 20 minutes

4.2 Why does blinking reduce eye strain?
☐ Spreads tears and prevents dryness
☐ Reduces brightness
☐ Exercises eye muscles
☐ Increases oxygen

4.3 Blue light mainly affects which function (especially at night)?
☐ Digestion ☐ Hearing ☐ Sleep ☐ Balance

4.4 Best screen adjustment to reduce eye strain
☐ Lowest contrast
☐ Smaller font
☐ Maximum brightness
☐ Adjust brightness appropriately + eye comfort mode

4.5 Most effective way to reduce eye fatigue
☐ Dim screen
☐ Take breaks
☐ Dark background
☐ Wear contact lenses

Section 5: Frequency and Severity of Digital Eye Strain Symptoms

Frequency

0 = Never
1 = Occasionally (~1 time/week)
2 = Frequently (≥2–3 times/week or daily)

Severity

1 = Mild–Moderate
2 = Severe

No.	Symptom
1	Burning eyes
2	Blurred vision
3	Eye pain
4	Frequent blinking
5	Itchy eyes
6	Headache
7	Tearing
8	Light sensitivity
9	Dry eyes
10	Reduced visual clarity
11	Difficulty focusing at near
12	Eye heaviness
13	Foreign body sensation
14	Double vision
15	Halos around lights
16	Red eyes

Total Score: ______

Thank you for your participation.
